# Allumo: Preprocessing and Calibration Software for Wearable Accelerometers Used in Posture Tracking

**DOI:** 10.3390/s20010229

**Published:** 2019-12-31

**Authors:** Alexis Fortin-Côté, Jean-Sébastien Roy, Laurent Bouyer, Philip Jackson, Alexandre Campeau-Lecours

**Affiliations:** 1 Center for Interdisciplinary Research in Rehabilitation and Social Integration, Quebec City, QC G1M 2S8, Canada; alexis.fortin-cote.1@ulaval.ca (A.F.-C.); Jean-Sebastien.Roy@fmed.ulaval.ca (J.-S.R.); Laurent.Bouyer@rea.ulaval.ca (L.B.); Philip.Jackson@psy.ulaval.ca (P.J.); 2 School of Psychology, Université Laval, Quebec City, QC G1V 0A6, Canada; 3 Department of Rehabilitation, Université Laval, Quebec City, QC G1V 0A6, Canada; 4 Department of Mechanical Engineering, Université Laval, Quebec City, QC G1V 0A6, Canada

**Keywords:** accelerometer, calibration, inertial measurement units, human movement

## Abstract

Inertial measurement units have recently shown great potential for the accurate measurement of joint angle movements in replacement of motion capture systems. In the race towards long duration tracking, inertial measurement units increasingly aim to ensure portability and long battery life, allowing improved ecological studies. Their main advantage over laboratory grade equipment is their usability in a wider range of environment for greater ecological value. For accurate and useful measurements, these types of sensors require a robust orientation estimation that remains accurate over long periods of time. To this end, we developed the Allumo software for the preprocessing and calibration of the orientation estimate of triaxial accelerometers. This software has an automatic orientation calibration procedure, an automatic erroneous orientation-estimate detection and useful visualization to help process long and short measurement periods. These automatic procedures are detailed in this paper, and two case studies are presented to showcase the usefulness of the software. The Allumo software is open-source and available online.

## 1. Introduction

Wearable sensors are increasingly being used in research and clinical practice to assess the pose and posture of individuals. For instance, physical rehabilitation may require objective movement measurements over extended periods of time to perform a comprehensive assessment of the patient. Being able to obtain quantitative measurements outside of controlled environments, such as a laboratory, through the use of wearable sensors could help in the diagnosis and treatment of patients. For instance, stride parameters are measured through GPS and inertial measurement unit (IMU) data [1], as well as gait and posture analyzed from pressure-sensitive insoles and IMU data [2]. Navigation estimates using IMUs with [3] and without GPS [4] have also been studied. IMU sensors have proven to be effective in orientation estimation, such as trunk orientation and lower limb kinematics [5] and in measuring the shoulder joint angles [6]. Accelerometers can also be used for impact detection and gait timing [7,8,9]. They have also been used in harsher conditions such as swimming [10]. As the number of contexts using these types of sensors increases, so does the need to improve orientation estimation accuracy. A static accuracy assessment of the Xsens IMU sensors [11] for 3D orientation positioning has been published [12]. Validation of the Xsens movement measurement [13] reported good correlation (0.96) between Xsens movement and vision-based measurements. Further assessment of accuracy for joint rotation for field-based occupational studies [14] reported measurement errors varying from $4^{\circ }$ to $12^{\circ }$. A recent systematic review reported that IMU should be considered as a valid tool to assess the whole body range of motion, and underlined the importance of the calibration step [15] to obtain such levels of accuracy.

Indeed, calibration is an important step to capture accurate pose estimates, especially for joint angle measurement [16]. The published procedure for pose calibration to align an IMU to another motion-capture system, which is presented in [17], and a pose calibration procedure for 3D knee joint angle [18]. Furthermore, Lotter et al. [19] published a procedure for in-use calibration of triaxial accelerometers that shares similarities with our proposed automatic orientation estimation algorithm, such as relying on the fact that the magnitude of the gravity acceleration vector measured with the accelerometer is constant and equals to 1g under quasi-static conditions. They use those assumptions to calibrate the tension readings of the triaxial acceleration (an offset and scaling for each axis). In our proposed automatic orientation algorithm, the same assumptions are leveraged to determine the orientation of the accelerometer reference frame with respect to the fixed, world reference frame. A review of several calibration methods of the former style for motion analysis is available in [20].

In the context of a project to collect mobility data, an important challenge occurs during the assessment of IMU data obtained in the field. Indeed, while the typical use case is a relatively short acquisition duration (minutes to hours long), larger scale mobility projects aim to record data over long periods of time. Corresponding time series data sets are thus quite large and require a tedious and time-consuming manual preprocessing task, during which the accuracy of the pose estimate can decrease. This concern was also raised in [21]. In mobility data, file duration can span over several weeks of continuous recording at 60 Hz (three data channels per accelerometer). While this data file size may not be considered large in a big data context, the amount of manual preprocessing required using the existing commercial tools precludes them from being used at the scale required for many projects. As participants remove and install the equipment, a manual recalibration of the orientation estimate of the devices is required and can be difficult in the field. Whereas some analyses such as automatic activity recognition does not require a known orientation [22], posture monitoring does, and therefore requires this recalibration step. Manual adjustments of the orientation estimate using only the data stream and without direct monitoring from an external observer in the field are impracticable. Furthermore, uncontrolled events such as unwanted shifts of the sensor on the participant may result in erroneous readings stemming from an inaccurate calibration. These also require identification and a subsequent sensor recalibration, which is also manually impracticable using the raw data stream. Identifying such erroneous readings is difficult with the available commercial tools, as they tend to only present time series plots of the data, a counter-intuitive method for detecting erroneous orientation estimate by most observers.

To help in the calibration of the orientation estimate of IMUs used for joint angle measurement, we present a tool for visualization and preprocessing to be used in human posture monitoring and assessment. The development goal of the software presented here was to expedite the identification and recalibration of the orientation estimate of triaxial accelerometer readings by showing an intuitive graphical interface to the observer. An animated humanoid avatar illustrates the estimated posture of the participant along the data stream. It makes it easier to identify erroneous orientation estimates since abnormal postures of the body will be displayed (e.g., wrong limb orientation, walking at a skewed angle).

This paper is structured as follows: first is the presentation and description of the automatic calibration of the orientation estimate, erroneous orientation-estimate detection and activity detection algorithms; second is an overview of the software followed by two case studies to show typical usage of the software.

## 2. Software Overview

**Figure 1:**
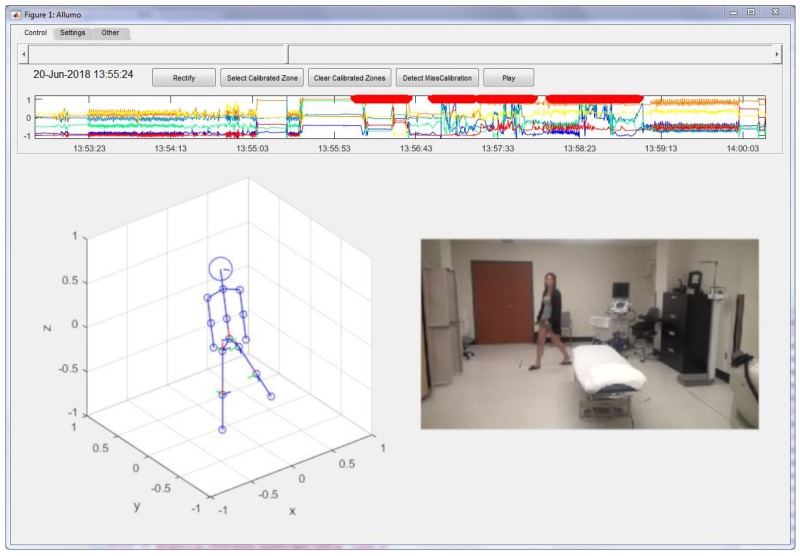
Main interface of the software.

The main software, of which the interface in presented in Figure 1, boasts several features useful for the preprocessing and assessment of IMU data. It features a real-time playback visualization of a humanoid model for easy diagnostics of improbable orientation estimate or wrongly positioned sensors. It helps to identify potential problems by displaying captured motion on an avatar model. For instance, a mis estimated orientation measurement could show a skewed trunk angle or unrealistic leg movement. It can also display a matching video to help with the visual comparison of the movement. Options in the settings are available to synchronize the video playback with the animated human shape motion. There is also a live display of the variables of interest, such as torso and leg joint angles, with respect to the vertical axis. For long duration data acquisitions, the software provides convenient time selection features that allow the definition of a specific working window.

The software also features an interface for manual selection of reference positions that is useful for the initialization of the orientation estimate of short recordings when reference points (neutral position) are known, e.g., a recording that begins with still sensors positioned in a known orientation. To improve the accuracy of the reference point measure, a section (window) of the signal can be marked as the reference orientation so that an averaging can be performed to reduce measurement noise during the reference position in the recording. To further help with this task, an algorithm, used for the automatic detection of erroneous orientation estimates, was implemented and is described in Section 4. Since manual adjustment of the initial orientation estimate can be complex and time-consuming, one of the software’s main features is the automatic calibration of the sensor initial orientation through automatic detection of motionless neutral positions (quasi-static), based on filtering and singular value decomposition, all of which are further described in Section 3. A basic automatic activity detection feature, used to distinguish between idling, walking and running, is also available.

Lastly, the software allows the importation of raw accelerometer values from multiple file formats such as Actigraph GT3x, comma-separated values (csv) files, and Excel spreadsheets. It allows the exportation of the calibrated accelerometer values to a convenient csv or Excel file format for uploading to most analytics programs.

## 3. Automatic Calibration Algorithm

**Figure 2:**
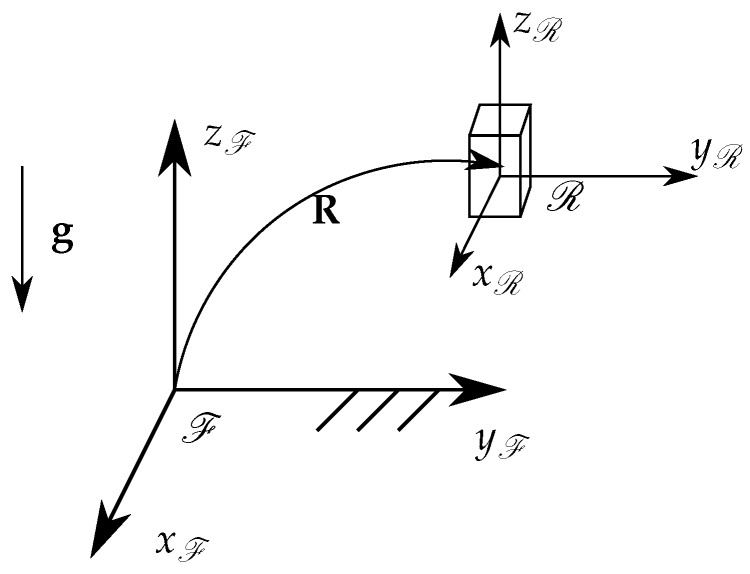
Geometric representation of the accelerometer.

We define the calibration of the orientation estimate of the triaxial accelerometer as "identifying the rotation matrix that aligns the mobile reference frame originating at the accelerometer with the fixed (world) reference frame". In other words, the orientation of the mobile reference frame R, with respect to the fixed reference frame F, is defined by the rotation matrix R as seen in Figure 2. This therefore defines the three rotational degrees of freedom (DOF) of the accelerometer. This allows for the full orientation of an arbitrarily placed sensor to be estimated. To this end, the automatic calibration algorithm leverages the gravitational force g, which is constant in the fixed reference frame, to constrain two of the three DOF, and a variance analysis to constrain the third DOF, fully defining the matrix R. The measured acceleration matrix is defined as

(1) $$A=\left[\begin{array}{ccc} a_{x1} & a_{y1} & a_{z1}\\ \vdots & \vdots & \vdots \\ a_{xm} & a_{ym} & a_{zm} \end{array}\right]\in R^{m\times 3},$$

where $a_{xi}$, $a_{yi}$, $a_{zi}$, are the $i^{\mathrm{th}}$ measurements along the $x_{R}$, $y_{R}$, $z_{R}$ axes respectively, and *m* is the index of the last measurement.

The automatic calibration algorithm works by using three main assumptions. First, when the participant is at rest, he/she is in the neutral position most of the time (e.g., standing upright). Second, the gravity vector can be measured without bias most of the time, meaning that no steady state acceleration should be present outside of gravity, i.e., the system is not in free fall or under centrifugal acceleration. Third, most of the variance in the movement occurs in the plane that is parallel to the gravitational force and in the forward-facing direction $x_{F}$ (e.g., walking). If these conditions are not met, a manual assisted option is available (see Section 4).

When those assumptions are observed, the algorithm works as follows. The first step is to find the direction of the gravitational force acting on the sensor. To this end, raw accelerometer data are filtered with a bidirectional low-pass filter with a cutoff frequency (COF) of 1/10 Hz to remove impacts, high-frequency noise and human limb movements to keep only low-frequency signals indicative of a steady state acceleration, which should correspond to a large extent to the neutral position. To accurately identify the direction of the gravity vector, the only data points kept for averaging are those where no movement is perceived (quasi-static). Those data points are found using a high-pass filter with the same COF of 1/10 Hz, filtering over the acceleration vector magnitude (l2 norm) instead of over each component, to identify where the signal lies closer to zero. A 2-s Hann window filter is used to further smooth the acceleration magnitude signal and keep only substantially long periods of quasi-static movements. The gravitational acceleration direction is then averaged across these sections with low movements. Knowing the unit vector $\hat{g}$, which represents the direction of the measured gravitational acceleration, a matrix $R^{\prime }$ such as

(2) $$g=R^{\prime }\hat{g},$$

that aligns the $z_{R}$ axis to the $z_{F}$ axis can be derived, therefore establishing two DOFs. To establish the last DOF, raw acceleration measurements are transformed with matrix $R^{\prime }$ so that the measured gravitational acceleration aligns with the negative $z_{F}$ direction, leaving acceleration on the $x_{R}$, $y_{R}$ planes as the only result of the accelerometer motion. The principal axis of motion is then computed using singular-value decomposition of the acceleration matrix A to find the largest singular value, $\sigma_{1}$, and its corresponding singular vector, $v_{1}$, such as

(3) A=UΣV,

with $U\in R^{m\times m}$, containing the left-singular vectors, $\Sigma \in R^{m\times 3}$ containing the singular values $\sigma_{1}\ldots \sigma_{3}$ and $V\in R^{3\times 3}$ containing the right-singular vectors including the vector of interest $v_{1}$. The singular vector $v_{1}$, which corresponds to the largest singular value, $\sigma_{1}$, is considered to be the forward-facing direction and therefore allows to establish the last DOF which, in turn, allows the full computation of R. Note that since the direction of the singular vector can be positive or negative, this leaves the distinction between forward and backward undefined, and is thus a known shortcoming of the method that needs to be corrected manually.

## 4. Automatic Erroneous-Orientation Detection

When the automatic orientation estimation calibration cannot be used (assumption not met), the automatic erroneous orientation-estimate detection provides an alternative assisted orientation estimation calibration option. It is based on the same assumption as the automatic calibration algorithm, but is only used as a tool for the detection of periods when manual orientation adjustment is necessary. The user can then manually discriminate between erroneous orientation estimates and other movements that may trigger the erroneous orientation-estimate detection, such as lying down for a long period of time. The algorithm operates by checking whether the gravitational acceleration aligns correctly with the negative $z_{F}$ direction. Using a bidirectional low-pass filter, it isolates the gravitational measurement, as described in Section 3, and flags sections of the accelerometer measurements in which the angle between the measured direction of gravity and the $z_{F}$ direction is larger than a user-defined threshold value. This way, the algorithm can help detect whether or not a sensor has moved since the last adjustment. A visual cue is presented to the user so that he/she can act on the flagged section by manually adding reference orientation point as presented in Section 2, or removing the section altogether.

## 5. Activity Detection

A basic activity detection algorithm is implemented in the software. It differentiates between three different states: idling, walking or running. Again, using a low-pass filter followed by a Hann window filter over the acceleration vector magnitude (l2 norm) generates an activity intensity signal. By using simple threshold values, the discrimination between idling, walking and running can be achieved.

## 6. Case Studies

### 6.1. Demonstration in the Laboratory

A participant wearing two Actigraph GT3X accelerometers, one placed on the left thigh and the other on his back, was asked to perform a 15-min routine with a mix of standing, walking, running and lying down. The activity was captured on video to qualitatively assess the pose estimates of reported by the software. During the routine, the orientation of the sensors on the body was deliberately altered at three different times to test the algorithm’s erroneous orientation-estimate detection. This resulted in 5 sections of signal being flagged using the erroneous orientation-estimate detection tool. The three deliberate orientation change and both time the participant lay down for an extended period of time had been correctly flagged. Using the playback of the humanoid visualization, the user can easily identify whether the detection is caused by an erroneous orientation estimate or a real change in steady-state operation such as going from a standing vertical to a horizontal orientation. This demonstration showed good performance in correctly identifying miscalibrated orientation estimates and was able to process the signal by applying the correct orientation adjustment parameters during each segment. With the correct adjustment applied, the user can look at the avatar and easily distinguish certain movements performed by the participant such as sitting, crouching and lying down. The activity detection algorithm was also able to correctly identify the two running events, the six walking events and the corresponding idling sequences between those.

### 6.2. Demonstration in the Field

In this data collection, the software has been used in the analysis of data from two expeditions lasting 39 and 32 days, respectively. During those expeditions, two participants wore two Actigraph GT3X accelerometers; one of the accelerometers was placed on the pelvis and the other on the left thigh. Both were affixed on shorts so that they moved along with the body. Data acquisition was performed over the 71 days for a total of around 1700 h of recording. Participants wore the sensors for the major part of the days and removed them during most sleep hours. Manual initialization of the orientation estimate for those types of extended periods is impractical. The automatic calibration algorithm was therefore used for each day of acquisition. After automatic calibration is performed, relevant data can be extracted, such as the amount of time walking or running and the trunk angle. The calibrated accelerometer’s data can then be exported for further processing. Table 1 shows an example of which data can be obtained for 1 hour segments of a day of recording and the corresponding logbook entries. By comparing the ratio of walking and running detection to corresponding entries, an overall assessment of the activity detection can be made. It can be seen that high trunk angle correlates with nap time and that running detection correlates with high-intensity activities. Walking proportion also varies sensibly with reported activities.

## 7. Conclusions

This paper presented a graphical software for preprocessing raw accelerometer data in the context of posture tracking. The software allows the visualization of measured posture on a humanoid form to easily identify errors in measurements or in the orientation estimate of the device in long duration, in the field, experiment. The paper also presented a novel algorithm for automatic calibration of the orientation estimate that can be used when manual initialization of the orientation estimate is impractical. A simple erroneous orientation-estimate detection and basic activity detection algorithms are implemented in the software. Two case studies were used to show typical usage of the software. The software is open-source and available at https://github.com/alexisfcote/allumo. 

## Figures and Tables

**Table 1:** Data summary samples for a 1-h expedition segment.

	Walking	Running	Trunk Angle	Logbook Entry
segment 1	7.40%	0.19%	$6.28^{\circ }$	Jumping jacks as a warm-up following by work at the computer
segment 2	7.20%	0.00%	$71.21^{\circ }$	Lying down for 50 min
segment 3	4.42%	0.00%	$3.53^{\circ }$	Work at the computer (mostly siting)
segment 4	12.78%	35.84%	$10.61^{\circ }$	30 min jogging followed by work at the laboratory
segment 5	6.89%	0.00%	$83.14^{\circ }$	50 min nap (lying down)
segment 6	17.08%	0.83%	$6.17^{\circ }$	Helicopter outing and walk ashore
segment 7	9.87%	0.02%	$5.47^{\circ }$	Diner and relaxation on board
